# Human Coronavirus Infections in Israel: Epidemiology, Clinical Symptoms and Summer Seasonality of HCoV-HKU1

**DOI:** 10.3390/v10100515

**Published:** 2018-09-21

**Authors:** Nehemya Friedman, Hadar Alter, Musa Hindiyeh, Ella Mendelson, Yonat Shemer Avni, Michal Mandelboim

**Affiliations:** 1 Central Virology Laboratory, Ministry of Health, Chaim Sheba Medical Center, Tel-Hashomer, P.O.B. 5265601, Ramat-Gan 5290002, Israel; Nehemya.Friedman@sheba.health.gov.il (N.F.); hadaralter1@gmail.com (H.A.); hindiyeh@yahoo.com (M.H.); Ella.Mendelson@sheba.health.gov.il (E.M.); 2 Department of Epidemiology and Preventive Medicine, School of Public Health, Sackler Faculty of Medicine, Tel-Aviv University, P.O.B. 39040, Tel-Aviv 69978, Israel; 3 Department of Microbiology, Immunology and Genetics, and the Clinical Virology Soroka University Medical Center, Faculty of Health Sciences, Ben Gurion University of the Negev, P.O.B. 653, Beer Sheva 84105, Israel; yonat@bgu.ac.il

**Keywords:** human coronavirus, HCoV-OC43, HCoV-NL63, HCoV-229E, HCoV-HKU1, Israel

## Abstract

Human coronaviruses (HCoVs) cause mild to severe respiratory diseases. Six types of HCoVs have been discovered, the most recent one termed the Middle East respiratory syndrome coronavirus (MERS-CoV). The aim of this study is to monitor the circulation of HCoV types in the population during 2015–2016 in Israel. HCoVs were detected by real-time PCR analysis in 1910 respiratory samples, collected from influenza-like illness (ILI) patients during the winter sentinel influenza survey across Israel. Moreover, 195 HCoV-positive samples from hospitalized patients were detected during one year at Soroka University Medical Center. While no MERS-CoV infections were detected, 10.36% of patients in the survey were infected with HCoV-OC43 (43.43%), HCoV-NL63 (44.95%), and HCoV-229E (11.62%) viruses. The HCoVs were shown to co-circulate with respiratory syncytial virus (RSV) and to appear prior to influenza virus infections. HCoV clinical symptoms were more severe than those of RSV infections but milder than influenza symptoms. Hospitalized patients had similar HCoV types percentages. However, while it was absent from the public winter survey, 22.6% of the patients were HCoV-HKU1 positives, mainly during the spring-summer period.

## 1. Introduction

Human coronaviruses (HCoVs), which are enveloped RNA viruses belonging to the Coronaviridae family, are associated with a wide spectrum of respiratory diseases [1,2]. HCoV infections occur mainly in the winter-spring season [3,4,5]. Thus far, six types of HCoV have been discovered in humans: HCoV-229E, HCoV-OC43, HCoV-NL63, HCoV-HKU1, severe acute respiratory syndrome coronavirus (SARS-CoV), and the Middle East respiratory syndrome coronavirus (MERS-CoV) [6].

HCoV-229E and HCoV-OC43 were first identified in 1967 as the cause of upper and mild respiratory tract infections [7,8]. During 2002 and 2003, SARS-CoV caused a worldwide epidemic, concluding in 8273 confirmed infections with a fatality rate of 9%; beside a few zoonotic cases and laboratory-acquired infections in December 2003 and in 2004, there have been no SARS-CoV transmitting within the human population after July 2003 [9,10]. SARS-CoV infection results in sudden onset of flu-like syndrome which includes fever, dry cough, and non-respiratory symptoms e.g., diarrhea, myalgia, headache and chills/rigors [11]. In 2004, HCoV-NL63 was isolated from a 7-month-old child suffering from bronchiolitis and conjunctivitis. Its distinctive genomic features were important in identifying seven additional HCoV-NL63-infected individuals suffering from respiratory illness [12]. In 2005, HCoV-HKU1 was isolated from patients with pneumonia and was defined as a new group of HCoV, featuring the lowest G + C content (32%) among all coronaviruses with a known genome sequence [13]. MERS-CoV was first identified in the Kingdom of Saudi-Arabia in September 2012 [14,15]. Dromedary camels are considered a possible source of MERS-CoV infection, since MERS-CoV neutralizing antibodies were found in camels from the Spanish Canary Islands, Oman, and Egypt [16]. Until April 2014, the World Health Organization (WHO) reported a total of 261 laboratory-confirmed MERS-CoV cases, resulting in 93 deaths. Of these, 145 were reported in Saudi Arabia and United Arab Emirates [17,18]. Recently a major MERS-CoV outbreak was reported in the Republic of Korea, with 186 infected individuals, resulting in 38 deaths [19]. At the end of May 2018, a total of 2220 laboratory-confirmed cases of MERS-CoV, including 790 associated deaths were reported globally; 1844 cases of these were reported from Saudi Arabia [20].

In this study, we analyze respiratory samples that were collected from patients presenting influenza-like illness (ILI) in a hospital and in the community in Israel during 2015–2016, for the presence of all known types of HCoV.

## 2. Materials and Methods

### 2.1. Clinical Samples

Nasopharyngeal samples were collected as part of the community influenza surveillance conducted in collaboration with the Israel Center for Disease Control (ICDC). The samples were collected from 1910 patients presenting with influenza-like illness (ILI), during the influenza season spanning between September 2015 and April 2016. Samples were obtained from over 26 outpatient clinics located in different geographical regions in Israel. In addition, 195 HCoV-positive samples from hospitalized patients were detected at Soroka University Medical Center, between July 2015 to June 2016.

### 2.2. Viral Genome Extraction and Real-Time PCR (qRT-PCR) Analysis

Viral genome was extracted from 500 µL of patient samples using the NucliSENS easyMAG kit (BioMerieux, Marcy-l’Étoile, France). Samples were tested for the presence of influenza viruses (A, B, and H1N1pdm) and respiratory syncytial virus (RSV), by qRT-PCR, as previously described [21,22]. To determine the human coronaviruses types: HCoV-NL63, HCoV-HKU1, HCoV-OC43, HCoV-229E, all samples were subjected to qRT-PCR, as previously described [23]. To determine the presence of MERS-CoV we tested by qRT-PCR assays for targeting regions upstream of the E gene (upE) [24]. The qRT-PCR reactions were performed in 25 µL Ag-Path Master Mix (Ambion, Life Technologies, Carlsbad, CA, USA), using TaqMan Chemistry with an ABI 7500 instrument (Foster city, CA, USA).

### 2.3. Ethical Considerations

The samples were obtained as part of influenza surveillance conducted in accordance with the Public Health Ordinance in Israel. The institutional review board (IRB) of the Sheba Medical Center approved this research, under Helsinki protocol number 4375-17-SMC (Date of approval, 19 September 2017).

### 2.4. Statistical Analysis

A Chi-square test was applied to evaluate the differences in percent positivity between the compared groups. A *p*-value < 0.05 was considered statistically significant. All analyses were performed using IBM^®^ SPSS^®^ Statistics software (Version 23, SPSS Inc., Chicago, IL, USA), SAS (SAS 9.1, SAS Institute Inc, Cary, NC, USA) and Excel software (Excel 2013 v15.0, Microsoft, Redmond, WA, USA).

## 3. Results

### 3.1. HCoV Prevalence in Israel during Winter 2015–2016

**Figure 1:**
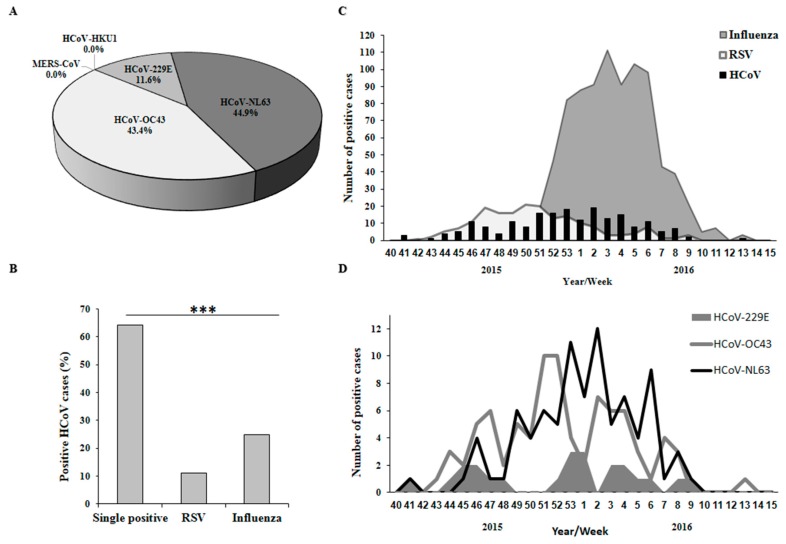
Respiratory viruses in the population during winter season 2015–2016. (**A**) Percentages of Human coronaviruses (HCoV) types. Pie chart presenting percentages of HCoV types among the 1910 influenza-like illness (ILI) samples collected in the 2015–2016 winter season; (**B**) Proportions of single and double-positive HCoV-positive samples. All samples were separated into three groups—HCoV, influenza, and respiratory syncytial virus (RSV). Each group was then separated to HCoV positive/negative. The bars represent the percentage of sample containing only HCoV (single positive) or co-infection with RSV or influenza. The chi-square statistic is 35.9447 and the *p*-value is < 0.00001; (**C**,**D**) Distribution of respiratory virus infection in the 2015–2016 winter season, starting from the 40th week of 2015 until the 15th week of 2016. (**C**) Distribution of HCoV, RSV and influenza viruses; (**D**) Distribution of HCoV types.

We analyzed samples of Influenza-like illness (ILI) patients in Israel for the presence of various viruses using qRT-PCR. We observed that 44.61% of the samples were positive for influenza viruses (of these, 56.34% were infected with influenza B, 42.96% with influenza A(H1N1)pdm09, 0.7% with influenza A(H3N2)) and 9.32% were positive for RSV. In addition, HCoV subtyping of the same patient samples demonstrated a 10.36% (*N* = 198) infection rate. Of these, 43.43% were infected with HCoV-OC43, 44.95% with HCoV-NL63 and 11.62% with HCoV-229E. MERS-CoV and HCoV-HKU1 were not detected (Figure 1A). Several samples showed multiple infections with HCoV and influenza or RSV (Figure 1B). There were no cases of co-infection with multiple HCoV types.

### 3.2. Weekly Distribution of HCoV, RSV and Influenza Infections

Next, we analyzed the weekly distribution of HCoVs, influenza viruses and RSV infections in 2015–2016. HCoVs and RSV showed similar patterns of infection, between October 2015 (week 42) and March 2016 (week 9) (Figure 1C), while influenza virus infection was noted primarily from December to February of the same period (Figure 1C). All HCoV sub-types distributed similarly during this time frame, whereas the highest number of infections for HCoV-OC43 were in weeks 51–52, for HCoV-NL63 was week 2 and for HCoV-229E weeks 53 and 1 (Figure 1D).

### 3.3. Age Distribution of Patients Carrying HCoV Infections

**Figure 2:**
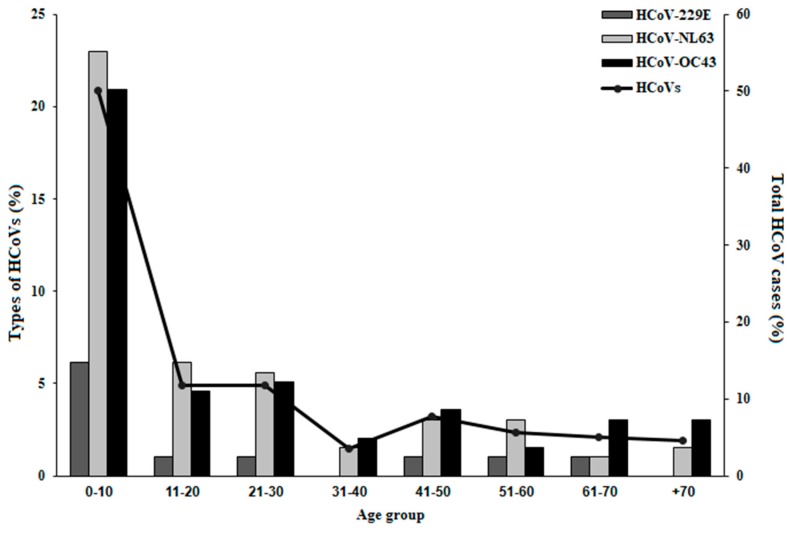
Age distribution of the HCoV-infected patients. The percent of all HCoV-positive cases (Line, Right Y-axis) and of each HCoV type (Bars, Left Y-axis) per patient age groups.

Influenza illness is especially problematic in the elderly, infants, and individuals with chronic diseases [25], while RSV is mainly hazardous in infants [26]. When assessing the age distribution of HCoV infection, we noted that HCoVs mainly infected infants and young children. More than fifty percent of HCoV-positive cases were in the 0–10 age group (Figure 2) while the remaining groups were around ten percent and below (Figure 2). HCoV-229E prevalence was low at all ages and absent in the 31–40 and 70+ age groups (Figure 2), probably due to its low prevalence compared to HCoV-OC43 and HCoV-NL63 (Figure 1A).

### 3.4. Clinical Symptoms of HCoV-Infected Individuals

**Figure 3:**
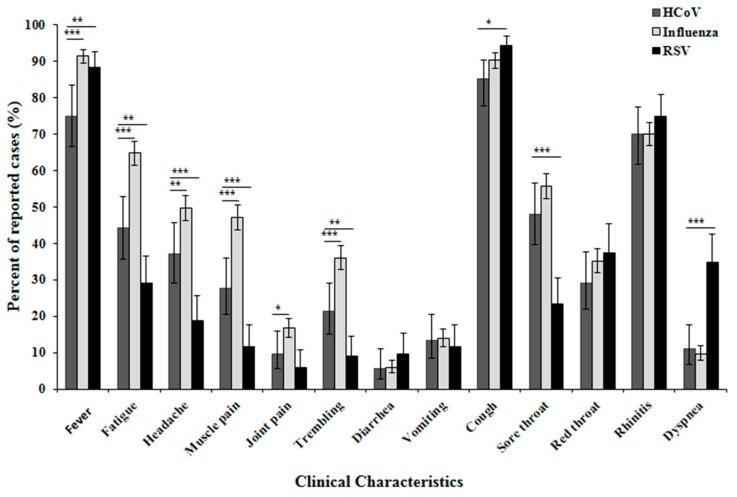
Clinical characteristics of patients infected with HCoV, RSV or influenza. A summary of the clinical symptoms of Israeli patients infected in the 2015–2016 winter season with HCoV, RSV or influenza. Confidence interval was calculated for the frequency of each symptom. */**/*** indicates *p*-value < 0.05/0.01/0.001 respectively, calculated in nonparametric Z-test for frequencies in IBM^®^ SPSS^®^ Statistics software (Version 23).

Analysis of the clinical symptoms reported by patients infected with influenza, RSV and HCoV, showed no significant differences in rates of diarrhea, vomiting, red throat and rhinitis cases between all viruses (Figure 3). In addition, the frequency of all symptoms assessed were similar or lower for HCoV infections compared to those of Influenza infections. Fewer HCoV patients had fever as compared to both influenza and RSV. More influenza-positive patients suffered from fatigue, headache, muscle pain, joint pain and trembling as compared to HCoV. Compared to RSV-infected patients, fever, cough and dyspnea were less frequent in HCoV-infected patients, while fatigue, headache, muscle pain, trembling and sore throat symptoms were more common among HCoV-infected patients (Figure 3). Other viruses responsible for upper respiratory tract infections were not analyzed (e.g., hPIV, hMPV, Rhinovirus, Adenoviruses, Bocaviruses).

### 3.5. Seasonality of HCoVs in Hospitalized Patients

**Figure 4:**
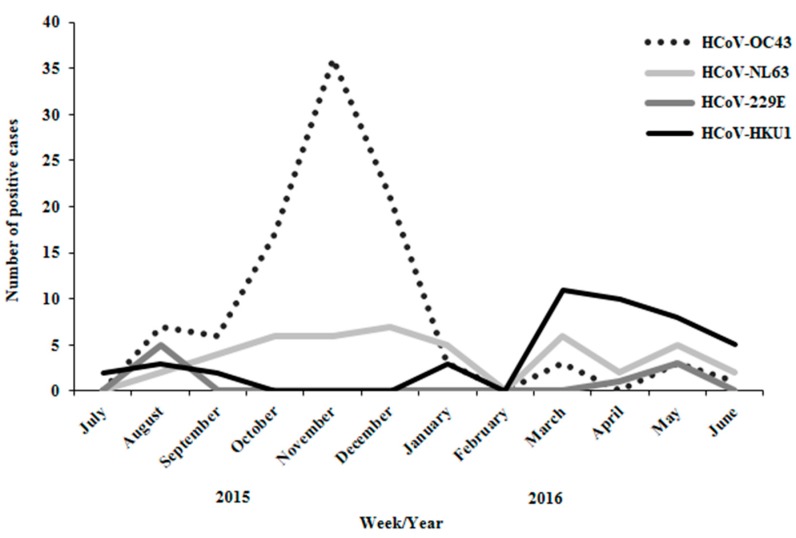
Year distribution of HCoV types in hospitalized patients. Months distribution of 195 hospitalized HCoV-positive cases from July 2015 until June 2016. Each HCoV type is presented as number of positive cases each month.

To get an insight about the seasonality of the viruses, we covered a full year survey on hospitalized patients since July 2015 to June 2016. Similar to the HCoV-positive cases in the community during the winter season, in hospitalized patients HCoV-OC43 (49.7%) and HCoV-NL63 (23.1%) constituted the majority while HCoV-229E (4.6%) was the minority. However, While HCoV-HKU1 was absent in the survey of the population, it infected many hospitalized patients (22.6% among HCoV-positive cases). HCoV-HKU1 was absent from our previous survey probably due to summer seasonality (Figure 4). In addition, HCoV-OC-43 was most prevalent during the winter months while HCoV-HKU1 was detected mainly in spring and summer months.

## 4. Discussion

Human coronaviruses 229E, NL63, OC43, and HKU1 infect people all over the world and usually cause mild to moderate upper-respiratory tract illnesses. SARS and MERS are respiratory infections which are exceptionally severe and sometimes may lead to death. No SARS-CoV infections have been reported since 2004, however, several outbreaks of MERS-CoV were noted in the recent years [27].

Here, we analyzed 1910 samples of ILI patients, collected in 26 sentinel clinics in Israel during winter season 2015–2016. Moreover, we examined 195 HCoV-positive samples from Soroka Medical Center, Beersheba, during one year. Approximately 50% of the samples in the public proved influenza- or RSV-positive. In addition, 198 HCoV-positive samples (10.36%) were identified, the majority carrying HCoV-OC43 or HCoV-NL63. Similarly, a study conducted in the Netherlands on healthy and hospitalized infants reported on dominancy of HCoV-OC43 and -NL63 infections [28]. Infections with HCoV-HKU1 or HCoV-229E were less frequent. The Dijkman R et al. hypothesis was that HCoV-OC43 and HCoV-NL63 may elicit immunity that protects from subsequent HCoV-HKU1 and HCoV-229E infections respectively [28]. Yet, morbidity associated with the various HCoV strains is inconsistent and subject to change from year to year [29]. In 2002–2003, a study in Bangkok, Thailand, found HCoV-229E and HCoV-OC43 viruses in 4.9% of acute respiratory tract illness samples of young children, with the majority being HCoV-229E infections [30]. In contrast, a study in rural Thailand found HCoV-OC43 to be dominant over HCoV-229E, suggesting that morbidity of the various types of HCoV infections also depends on regional environment [29]. Analysis of the prevalence of coronaviruses in children hospitalized with fever and acute respiratory symptoms in Hong Kong during 2001–2002, showed that HCoV-NL63 was dominant (57.69% of HCoV-positive samples), followed by HCoV-OC43 (34.61%) and HCoV-229E (7.69%) [31]. A retrospective study in Australia tested for four HCoV types in the winter season of 2004. A total of 74 coronavirus infections (8.3%) were detected, with HCoV-HKU1 being the most dominant (46.6% of all HCoV), followed by HCoV-OC43 (37.0%), HCoV-NL63 (12.3%) and HCoV-229E (5.5%) [32]. These findings, together with similar analyses by other groups [29,30,31,33,34,35] demonstrate that morbidity associated with the various HCoV types is difficult to predict since they do not infect every year, their infection rate varies and depends on geographical environment. In general, HCoV-OC43, HCoV-229E and HCoV-NL63 have been reported to have winter seasonality, however, in Hong Kong HCoV-NL63 had a spring-summer peak of activity [12,31,36]. In our study, HCoV-229E, HCoV-OC43 and HCoV-NL63 co-circulated with RSV throughout the entire winter season, starting before and persisting until the end of the influenza season. On the other hand, HCoV-HKU1 circulates in the spring-summer season. Generally, HCoVs co-circulate and display marked winter seasonality between the months of December and April and are not detected in summer months [37]. To our knowledge, this is the first time HCoV-HKU1 is reported to circulate in summer season. Environmental conditions may contribute to understand this finding. During December 2015 until February 2016 and April 2016 until June 2016, the average temperature and humidity in Israel were 19 °C, 57% and 25 °C, 66% respectively (An average data from Tel-Aviv station #2410 at 2 pm, collected from Israel Meteorological Service database, https://ims.data.gov.il/ims).

Coronaviruses infect individuals at all ages, as previously reported [38,39], causing mild to severe respiratory diseases [1]. This determination is consistent with our findings; however, we noted that more than 50% of the HCoV-positive patients were children. A major seroconversion for HCoVs occurs in infants up to the age of 20 months and immunity against one HCoV may influence future infection by other HCoV type [28]. Israeli HCoVs were associated with significantly lower or similar symptoms frequencies as compared to influenza. Yet, HCoV-229E, HCoV-OC43 and HCoV-NL63 patients had higher symptom frequencies for fatigue, headache, muscle pain, trembling and sore throat as compared to RSV patients. This observation may be of importance when considering hospitalization of infants and young children, that are more susceptible to RSV infection [26], especially when HCoVs and RSV co-circulate.

Despite its circulation in the Middle East and recent eruption in South-Korea, no MERS-CoV has been reported in Israel since its first detection in Saudi-Arabia in 2012 [14,19]. However, other types of HCoV were detected during the 2015–2016 winter (HCoV-OC43, -NL63 and -229E) and spring-summer (HCoV-HKU1) seasons. It is difficult to predict the combination and dominancy of each HCoV type for the next season.

## References

[1] Geller C., Varbanov M., Duval R.E. (2012). Human coronaviruses: Insights into environmental resistance and its influence on the development of new antiseptic strategies. Viruses.

[2] Razuri H., Malecki M., Tinoco Y., Ortiz E., Guezala M.C., Romero C., Estela A., Breña P., Morales M.L., Reaves E.J. (2015). Human Coronavirus-Associated Influenza-Like Illness in the Community Setting in Peru. Am. J. Trop. Med. Hyg..

[3] Gerna G., Campanini G., Rovida F., Percivalle E., Sarasini A., Marchi A., Baldanti F. (2006). Genetic variability of human coronavirus OC43-, 229E-, and NL63-like strains and their association with lower respiratory tract infections of hospitalized infants and immunocompromised patients. J. Med. Virol..

[4] Gerna G., Percivalle E., Sarasini A., Campanini G., Piralla A., Rovida F., Genini E., Marchi A., Baldanti F. (2007). Human respiratory coronavirus HKU1 versus other coronavirus infections in Italian hospitalised patients. J. Clin. Virol..

[5] Vabret A., Dina J., Gouarin S., Petitjean J., Tripey V., Brouard J., Freymuth F. (2008). Human (non-severe acute respiratory syndrome) coronavirus infections in hospitalised children in France. J. Paediatr. Child Health.

[6] King A.M., Lefkowitz E., Adams M.J., Carstens E.B. (2011). Virus Taxonomy: Ninth Report of the International Committee on Taxonomy of Viruses.

[7] Almeida J.D., Tyrrell D.A. (1967). The morphology of three previously uncharacterized human respiratory viruses that grow in organ culture. J. Gen. Virol..

[8] Hamre D., Procknow J.J. (1966). A new virus isolated from the human respiratory tract. Proc. Soc. Exp. Biol. Med..

[9] Drosten C., Günther S., Preiser W., van der Werf S., Brodt H.R., Becker S., Rabenau H., Panning M., Kolesnikova L., Fouchier R.A. (2003). Identification of a novel coronavirus in patients with severe acute respiratory syndrome. N. Engl. J. Med..

[10] Chinese SARS Molecular Epidemiology Consortium (2004). Molecular evolution of the SARS coronavirus during the course of the SARS epidemic in China. Science.

[11] WHO Guidelines for the Global Surveillance of Severe Acute Respiratory Syndrome (SARS) Updated Recommendations, October 2004; 2015. http://www.who.int/csr/resources/publications/WHO_CDS_CSR_ARO_2004_1/en/.

[12] Van der Hoek L., Pyrc K., Jebbink M.F., Vermeulen-Oost W., Berkhout R.J., Wolthers K.C., Wertheim-van Dillen P.M., Kaandorp J., Spaargaren J., Berkhout B. (2004). Identification of a new human coronavirus. Nat. Med..

[13] Woo P.C., Lau S.K., Chu C.M., Chan K.H., Tsoi H.W., Huang Y., Wong B.H., Poon R.W., Cai J.J., Luk W.K. (2005). Characterization and complete genome sequence of a novel coronavirus, coronavirus HKU1, from patients with pneumonia. J. Virol..

[14] Zaki A.M., van Boheemen S., Bestebroer T.M., Osterhaus A.D., Fouchier R.A. (2012). Isolation of a novel coronavirus from a man with pneumonia in Saudi Arabia. N. Engl. J. Med..

[15] Hijawi B., Abdallat M., Sayaydeh A., Alqasrawi S., Haddadin A., Jaarour N., Alsheikh S., Alsanouri T. (2013). Novel coronavirus infections in Jordan, April 2012: Epidemiological findings from a retrospective investigation. East. Mediterr. Health J..

[16] Haagmans B.L., Al Dhahiry S.H., Reusken C.B., Raj V.S., Galiano M., Myers R., Godeke G.J., Jonges M., Farag E., Diab A. (2014). Middle East respiratory syndrome coronavirus in dromedary camels: An outbreak investigation. Lancet Infect. Dis..

[17] Brown C. (2014). MERS differs from SARS, say experts. CMAJ.

[18] Center for Disease Control and Prevention (2017). Middle East Respiratory Syndrome (MERS).

[19] Kim K.H., Tandi T.E., Choi J.W., Moon J.M., Kim M.S. (2017). Middle East respiratory syndrome coronavirus (MERS-CoV) outbreak in South Korea, 2015: Epidemiology, characteristics and public health implications. J. Hosp. Infect..

[20] EMRO/WHO MERS Situation Update, May 2018. http://www.emro.who.int/pandemic-epidemic-diseases/mers-cov/mers-situation-update-may-2018.html.

[21] Hindiyeh M., Levy V., Azar R., Varsano N., Regev L., Shalev Y., Grossman Z., Mendelson E. (2005). Evaluation of a multiplex real-time reverse transcriptase PCR assay for detection and differentiation of influenza viruses A and B during the 2001–2002 influenza season in Israel. J. Clin. Microbiol..

[22] Meningher T., Hindiyeh M., Regev L., Sherbany H., Mendelson E., Mandelboim M. (2014). Relationships between A(H1N1)pdm09 influenza infection and infections with other respiratory viruses. Influenza Other Respir. Viruses.

[23] Lieberman D., Shimoni A., Keren-Naus A., Steinberg R., Shemer-Avni Y. (2009). Identification of respiratory viruses in adults: Nasopharyngeal versus oropharyngeal sampling. J. Clin. Microbiol..

[24] Corman V.M., Eckerle I., Bleicker T., Zaki A., Landt O., Eschbach-Bludau M., van Boheemen S., Gopal R., Ballhause M., Bestebroer T.M. (2012). Detection of a novel human coronavirus by real-time reverse-transcription polymerase chain reaction. Eurosurveillance.

[25] Taubenberger J.K., Kash J.C. (2010). Influenza virus evolution, host adaptation, and pandemic formation. Cell Host Microbe.

[26] Glezen W.P., Taber L.H., Frank A.L., Kasel J.A. (1986). Risk of primary infection and reinfection with respiratory syncytial virus. Am. J. Dis. Child..

[27] Centers for Disease Control and Prevention About Coronavirus. 22 August 2016. https://www.cdc.gov/coronavirus/about/index.html.

[28] Dijkman R., Jebbink M.F., Gaunt E., Rossen J.W., Templeton K.E., Kuijpers T.W., van der Hoek L. (2012). The dominance of human coronavirus OC43 and NL63 infections in infants. J. Clin. Virol..

[29] Dare R.K., Fry A.M., Chittaganpitch M., Sawanpanyalert P., Olsen S.J., Erdman D.D. (2007). Human coronavirus infections in rural Thailand: A comprehensive study using real-time reverse-transcription polymerase chain reaction assays. J. Infect. Dis..

[30] Theamboonlers A., Samransamruajkit R., Thongme C., Amonsin A., Chongsrisawat V., Poovorawan Y. (2007). Human coronavirus infection among children with acute lower respiratory tract infection in Thailand. Intervirology.

[31] Chiu S.S., Chan K.H., Chu K.W., Kwan S.W., Guan Y., Poon L.L., Peiris J.S. (2005). Human coronavirus NL63 infection and other coronavirus infections in children hospitalized with acute respiratory disease in Hong Kong, China. Clin. Infect. Dis..

[32] Mackay I.M., Arden K.E., Speicher D.J., O’Neil N.T., McErlean P.K., Greer R.M., Nissen M.D., Sloots T.P. (2012). Co-circulation of four human coronaviruses (HCoVs) in Queensland children with acute respiratory tract illnesses in 2004. Viruses.

[33] Esper F., Weibel C., Ferguson D., Landry M.L., Kahn J.S. (2005). Evidence of a novel human coronavirus that is associated with respiratory tract disease in infants and young children. J. Infect. Dis..

[34] Lau S.K., Woo P.C., Yip C.C., Tse H., Tsoi H.W., Cheng V.C., Lee P., Tang B.S., Cheung C.H., Lee R.A. (2006). Coronavirus HKU1 and other coronavirus infections in Hong Kong. J. Clin. Microbiol..

[35] Bastien N., Anderson K., Hart L., Van Caeseele P., Brandt K., Milley D., Hatchette T., Weiss E.C., Li Y. (2005). Human coronavirus NL63 infection in Canada. J. Infect. Dis..

[36] Vabret A., Mourez T., Gouarin S., Petitjean J., Freymuth F. (2003). An outbreak of coronavirus OC43 respiratory infection in Normandy, France. Clin. Infect. Dis..

[37] Gaunt E.R., Hardie A., Claas E.C., Simmonds P., Templeton K.E. (2010). Epidemiology and clinical presentations of the four human coronaviruses 229E, HKU1, NL63, and OC43 detected over 3 years using a novel multiplex real-time PCR method. J. Clin. Microbiol..

[38] Vabret A., Brouard J., Petitjean J., Eugene-Ruellan G., Freymuth F. (1998). Human coronavirus infections: Importance and diagnosis. Presse Med..

[39] Van der Hoek L., Pyrc K., Berkhout B. (2006). Human coronavirus NL63, a new respiratory virus. FEMS Microbiol. Rev..

